# Appropriate Safeguards and Article 89 of the GDPR: Considerations for Biobank, Databank and Genetic Research

**DOI:** 10.3389/fgene.2022.719317

**Published:** 2022-02-18

**Authors:** Ciara Staunton, Santa Slokenberga, Andrea Parziale, Deborah Mascalzoni

**Affiliations:** ^1^ Institute for Biomedicine, Eurac Research, Bolzano, Italy; ^2^ School of Law, Middlesex University, London, United kingdom; ^3^ Faculty of Law, Uppsala University, Uppsala, Sweden; ^4^ Department of Business and Management, Luiss Guido Carli University, Rome, Italy; ^5^ Center for Research Ethics and Bioethics, Department of Public Health and Caring Sciences, Uppsala University, Uppsala, Sweden

**Keywords:** GDPR–general data protection regulation, biobank, genetic research, safeguards, consent, ethics review and governance

## Abstract

The collection and use of biological samples and data for genetic research, or for storage in a biobank or databank for future research, impacts upon many fundamental rights, including the right to dignity, the right to private and family life, the right to protection of personal data, the right to freedom of arts and sciences, and the right to non-discrimination. The use of genetic data and other health-related data in this context must be used in a manner that is rooted in human rights. Owing in part to the General Data Protection Regulation (GDPR) coming into force, the right to the protection of personal data in the context of scientific research has been afforded increasing attention. The GDPR gives effect to the right to data protection, but states that this right must be balanced against other rights and interests. The GDPR applies to all personal data, with specific attention to special categories of data, that includes health and genetic data. The collection, access to, and sharing of such data must comply with the GDPR, and therefore directly impacts the use of such data in research. The GDPR does provide for several derogations and exemptions for research from many of the strict processing requirements. Such derogations are permitted only if there are appropriate safeguards in place. Article 89 states that to be appropriate, safeguards must be “in accordance” with the GDPR “for the rights and freedoms of the data subject”. In particular, those safeguards must ensure “respect for the principle of data minimisation”. Despite the importance of safeguards, the GDPR is silent as to the specific measures that may be adopted to meet these requirements. This paper considers Article 89 and explores safeguards that may be deemed appropriate in the context of biobanks, databanks, and genetic research.

## Introduction

Genetic research requires access to large quantities of biological samples and data that can be collected directly from a research participant (research participant is used to describe a person from whom data and samples have been collected and includes the term “data subject”), or provided by a biobank or databank. This data is considered to be sensitive (Slokenberga, 2021), and the use of such data in research, touches on many of our fundamental rights. Under the Charter of the Fundamental Rights of the European Union, these include human dignity (Article 1), the right to integrity (Article 3), the right to respect for private and family life (Article 7), the right to protection of personal data (Article 8), freedom of arts and sciences (Article 13), and the right to non-discrimination (Article 21). Due in part to the adoption of the General Data Protection Regulation (GDPR), it is the right to data protection and its impact on health research that has been given increasing attention. The GDPR seeks to give effect to the right to data protection, but, in Recital 4, it states that this right must be balanced against other fundamental rights. The GDPR, thus, puts the right to data protection in the broader context of fundamental rights and in considering the protection of personal data in the context of genetic and biobank research, one needs to be cognizant of the other rights that are engaged.

The GDPR sets out the principles and rights that must be met in the processing of personal data, including the processing of personal data for research. These principles are lawfulness, fairness and transparency; purpose limitation; data minimization; accuracy; storage limitation; security; accountability. Meeting many of these principles, notably the principle of purpose limitation, data minimization, and storage limitation, can be challenging in the context of scientific research. The GDPR anticipated many of these challenges and affords certain privileges for research, through the provision of a framework for the derogation of some of the rights and processing requirements when the processing is for scientific research. This can either be done by directly invoking provisions of the GDPR itself, or through Member State or EU law.

If one first looks at the derogations provided for within the text of the GDPR itself, the principles of purpose limitation and storage limitation can be exempted from if the processing is for research purposes (Article 5(1)(c) and 5(1)(e)). Thus, personal data may be retained for longer than necessary and used for a purpose not specified at data collection if it is to be used for research. Interestingly, however, Article 6 GDPR does not assign a specific legal basis for scientific research to enable this to happen in a smooth manner. The right to information (Article 14), the right to erasure (Article 17), and the right to object (Article 21) can be exempted from under the direct applicability of the regulation, in line with Article 288 Treaty on the Functioning of the European Union (TFEU), if the processing is for research purposes and if the obligation of that right is likely to render impossible or seriously impair the research. The exact meaning of “impossible or seriously impair the achievement of the objectives” in the research context is beyond the scope of this article. However, it would appear that this needs to be considered on a case-by-case basis, and such impairments are more than tolerable inconveniences.

Now turning to the second framework for research, specific laws could provide for derogations for research purposes. Although the processing of special categories of personal data, that includes health and genetic data, is generally prohibited, it is permitted if (amongst other grounds outlined in Article 9) the processing is for research purposes outlined in EU or Member State law (Article 9(2)(j)). These laws can provide for derogations from the right of access (Article 15), right to rectification (Article 16), right to restriction of processing (Article 18), and right to object (Article 21), if these rights are likely to render impossible or seriously impair the research.

There are limits to these derogations that need to be read in light of the general rule in EU law that rules establishing exceptions shall be narrowly interpreted (Staunton et al., 2019). Specifically, reliance on the derogations are only valid if the following conditions are met: 1) the processing is for research; 2) reliance on the derogations is necessary to fulfil the objectives of the research; and 3) the derogations comply with the requirements outlined in Article 89(1) (for derogations and exemptions contained within the GDPR) or Article 89(2) (for derogations and exemptions set out in law). Moreover, and in line with proportionality as one of the general principles of EU law, they need to be able to pass the general requirement of suitability, necessity, and proportionality. It is the requirements contained within Article 89 that are the subject of this paper.

### Legitimate Safeguards Under Article 89

Article 89 requires that any derogations, irrespective of whether provided for by national law or EU law or by the GDPR itself, are subject to appropriate safeguards to protect the rights and freedoms of the data subjects. Article 89(1) states:

“Processing for archiving purposes in the public interest, scientific or historical research purposes or statistical purposes, shall be subject to appropriate safeguards, in accordance with this Regulation, for the rights and freedoms of the data subject. Those safeguards shall ensure that technical and organizational measures are in place in particular in order to ensure respect for the principle of data minimization.”

Derogating from any rights or principles are therefore not legitimate unless the necessary safeguards are in place. We have previously noted that reliance on the possible derogations contained within the GDPR could, in some situations, leave the research participant with almost no individual rights afforded under Chapter III of the GDPR if the processing is for research (Staunton et al., 2019). Therefore, these safeguards are crucial in protecting the rights and interests of the research participant.

The GDPR itself provides limited guidance as to the specific safeguards that are to be adopted. Article 89(1) refers to “technical and organizational safeguards” and states that personal data should be pseudonymised where possible and Recital 33 states that data subjects should be permitted to give their consent in line with ethical standards for scientific research. The European Data Protection Board (EDPB) has acknowledged that the scope of Article 89(1) and the safeguards require further clarification, and such clarification will be provided in the much anticipated EDPB Guidelines on processing personal data for scientific research purposes (EDPB, 2020b). The European Health Data Space (EHDS) is also being developed and it seeks to build a system of data governance and rules on data exchange that includes the use of data in research. The EHDS should also provide some clarity on possible safeguards. In the meantime, data controllers who are directly invoking the derogations contained within the GDPR or, as specified in Recital 156, by Member States who are providing for derogations under Article 89(2), must identify what they consider to be the safeguards when they seek to rely on the derogations.

As discussed, Article 89 does not specify the safeguards, but it does provide several features of the safeguards. They must be *appropriate*, they must be *in accordance with the GDPR*, and these safeguards must ensure that technical and organizational measures are in place to ensure *respect for the principle of data minimization*. Thus, the legitimacy of the safeguards in a particular context is contingent on them *all* being appropriate for the particular processing purpose; that *all* safeguards adopted are in accordance with the GDPR and for the rights and freedoms of the data subject; and that *some* of the safeguards must ensure the respect for the principle of data minimization. The meaning of “in accordance” requires some unpacking.

In the field of biobank research specifically, and the use of data in research more broadly, several regulatory instruments have been developed to protect the research participant, protect their rights, and ensure the ethical conduct of research. Arguably data controllers, national law or EU law could adopt the various rules and procedures identified in these instruments as safeguards under Article 89 as they are aimed at (amongst others) safeguarding the rights and interests of the research participant. Adopting these instruments would incorporate bioethical rules and procedures and thereby provide an integrated bioethics approach to data protection in the field of research, but consideration must be given to whether such instruments can be considered to be “in accordance” with the GDPR.

Of central importance in discussing the meaning of the wording “in accordance” with the GDPR is the objective of the regulation. At its core, the GDPR is about facilitating the flow of data in a manner that protects fundamental rights and freedoms, particularly the right to data protection. In the research context, the GDPR is not seeking to frustrate or disrupt research. Rather, it aims to ensure the use of data in research while protecting the rights and freedoms of the data subjects (Article 1(2)). However, in considering what is “in accordance” with this aim, there are two possible interpretations.

A narrow reading would limit possible safeguards to those that are in accordance with the principles and rights of the GDPR. The EDPB’s initial discussions on safeguards could point to such an interpretation. In its Preliminary Opinion on Data Protection and Scientific Research, the EDPB has taken a close reading of possible safeguards as generally coming directly from the GDPR (EDPB, 2020b). It notes that appropriate safeguards could include conducting a data protection impact assessment of likely risks, appointing a data protection officer, notifying data subjects of a data breach, guaranteeing data security, and data minimization through pseudonymisation or anonymization. These are all safeguards that are already expressly stated within the GDPR and focused on giving effect to the right to data protection in the research context. Informed consent, access limitations, and professional ethical standards are also highlighted by the EDPB as possible safeguards in the research context, but they are similarly provided for within the GDPR and its recitals.

The EDPB Guidelines on the processing of data concerning health for scientific research in the context of the COVID-19 outbreak, are quite similar. In addition to pseudonymisation, the EDPB stated that the safeguards should *at least* include encryption, non-disclosure agreements and strict access role distribution, access role restrictions as well as access logs (EDPB, 2020a). Again, these safeguards are primarily aimed at protecting personal data, perhaps indicating a narrow reading of safeguards.

A second wider reading would look at the right to data protection in the broader context of fundamental rights. In the research context, as noted earlier, the right to data protection is only one of a number of rights that are engaged. Research does occupy a privileged position under the GDPR and the right to freedom of arts and sciences under Article 13 of the Charter of Fundamental Rights of the EU (CFREU) is clearly engaged. However, Recital 4 of the GDPR states that it is “designed to serve mankind” and in the context of scientific research during COVID, the EDPB has stated that neither the Data Protection Rules nor the right to freedom of science under Article 13 “have precedence over the other”, but requires a balancing of these rights. However, what of the other rights discussed above? Does Article 89 enable the use of safeguards that may not directly be aimed at safeguarding the protection of data, but seeking to safeguard other fundamental rights as recognized by the GDPR? This question will be particularly pertinent in situations where a safeguard is aimed at protecting the research participant generally, or indeed the wider community (as distinct from data protection) and introduce barriers that either limits or prevents the use of personal data in research. For example, community engagement is increasingly seen as an important part in the ethical conduct of research (Tindana et al., 2015). If, as a result of engagement with the community, research or the sharing of data for research is either stalled, limited, or prevented from going ahead, could that be deemed to be a safeguard?

One could echo the argument that, although the GDPR is an important contribution to the research regulatory fora, it is not a self-sufficient research regulatory instrument (Slokenberga, 2021). Recital 156 seems to acknowledge that it is not directly responsible for regulating research through its “hands-off” clause as it states that “processing of personal data for scientific purposes should also comply with other relevant legislation such as on clinical trials”. On the other hand, Article 1(2) underlines that the regulation protects fundamental rights, Recital 4 specifically states that this right to data protection must be balanced with other rights, and Article 89 specifically stresses the “rights and freedoms” of the data subject in the context of research. Furthermore, the EU tends to regulate indirectly through the “back-door” approach, i.e., contributing to shaping the areas where it does not have direct competence through the areas where it has competency. It is therefore not precluded that the GDPR research regime could be used as a tool to reshape the existing research standards in biobanking.

Any final decision on the extent to which appropriate safeguards that are found in different research regulatory instruments are “in accordance” with the GDPR will of course rest with the Court of Justice of the EU (CJEU) under its exclusive competence to deliver authoritative interpretation of the EU law. For now, clarification from the EDPB on this point in the short-term and from the EHDS in the medium-term would be very much welcomed.

In this paper, we consider appropriate safeguards in the context of biobank research and genetic research. This paper builds upon our 2019 study where we reviewed 18 legal and ethical instruments that regulate health research and noted that reliance on all of the possible research derogations may be in breach of ethical guidance and best practice (Staunton et al., 2019). We now draw upon those instruments to first identify possible safeguards that can be drawn from these instruments. Second, we analyze these safeguards under the three factors set out above that are required for legitimate safeguards: 1) they are appropriate; 2) they are in accordance with the GDPR for the rights and freedoms of the data subject; 3) some ensure the respect for the principle of data minimization.

## Methods

We re-examined the ethical guidance, legal tools, international treaties and other legal instruments (hereinafter collectively called ‘instruments’) that were identified in our 2019 study (see Table 1). The 2019 Council of Europe Recommendation, published after the conclusion of the 2019 were identified. CS, AP, and DM examined these instruments to determine the instruments that contained guidance on possible safeguards and 5 instruments were removed as they did not provide any guidance on possible safeguards: European Convention on Human Rights, International Convention on Economic, Social and Cultural Rights, and the universal Declaration on Human Rights. The GDPR was also removed as the purpose of this paper was to identify possible safeguards from instruments other than within the GDPR. The full list of all instruments are in Table 2.

**TABLE 1:** instruments from 2019 study.

	Instrument	Year	Issuing body
1	GDPR	2016	European Union
2	Charter of Fundamental Rights and Freedoms of the European Union	2000	European Union
3	European Convention on Human Rights	1953	Council of Europe
4	Convention for the Protection of Human Rights and Dignity of the Human Being with regard to the Application of Biology and Medicine: Convention on Human Rights and Biomedicine (Oviedo Convention)	1997	Council of Europe
5	Convention for the protection of individuals with regard to the processing of personal data	1980 (revised 2018)	Council of Europe
6	Recommendation CM/Rec (2016)6 of the Committee of Ministers to member States on research on biological materials of human origin	2016	Council of Europe
7	Additional protocol to the Convention on Human Rights and Biomedicine, concerning Biomedical Research	2005	Council of Europe
8	International Convention on Economic, Social and Cultural Rights	1966	United Nations
9	Universal Declaration on Human Rights	1948	United Nations
10	Universal Declarationon Bioethics and Human Rights	2005	UNESCO
11	International Declaration on Human Genetic Data	2003	UNESCO
12	Universal Declaration on the Human Genome and Human Rights	1997	UNESCO
13	Recommendation on Health Data Governance	2017	OECD
14	OECD Guidelines on Human Biobanks and Genetic Research Databases	2009	OECD
15	Principles and Guidelines for Access to Research Data from Public Funding	2007	OECD
16	Declaration of Helsinki	2013 edition	World Medical Association
17	Declaration of Taipei on Ethical Considerations Regarding Health Databases and Biobanks	2002 (revised 2016)	World Medical Association
18	International Ethical Guidelines for Health-related Research Involving Humans	2016	Council for International Organizations of Medical Sciences

**TABLE 2:** instruments in current study.

	Instrument	Year	Issuing body
1	Convention for the Protection of Human Rights and Dignity of the Human Being with regard to the Application of Biology and Medicine: Convention on Human Rights and Biomedicine (Oviedo Convention)	1997	Council of Europe (Ovideo)
2	Convention for the protection of individuals with regard to the processing of personal data	1980 (revised 2018)	Council of Europe (2018 CoE)
3	Recommendation CM/Rec (2016)6 of the Committee of Ministers to member States on research on biological materials of human origin	2016	Council of Europe (2016 CoE)
4	Additional protocol to the Convention on Human Rights and Biomedicine, concerning Biomedical Research	2005	Council of Europe (2005 CoE)
5	Recommendation on the protection of health-related data	2019	Council of Europe (2019 CoE)
6	Universal Declaration on Bioethics and Human Rights	2005	UNESCO (2005 UNESCO)
7	International Declaration on Human Genetic Data	2003	UNESCO (2003 UNESCO)
8	Universal Declaration on the Human Genome and Human Rights	1997	UNESCO (1997 UNESCO)
9	Recommendation on Health Data Governance	2017	OECD (2017 OECD)
10	OECD Guidelines on Human Biobanks and Genetic Research Databases	2009	OECD (2009 OECD)
11	Principles and Guidelines for Access to Research Data from Public Funding	2007	OECD (2007 OECD)
12	Declaration of Helsinki	2013 edition	World Medical Association (Helsinki)
13	Declaration of Taipei on Ethical Considerations Regarding Health Databases and Biobanks	2002 (revised 2016)	World Medical Association (Taipei)
14	International EthicalGuidelines for Health-related Research Involving Humans	2016	Council for International Organizations of Medical Sciences (CIOMS)

CS initially reviewed these instruments and identified requirements that intended to protect research participants. Based on this review, a code book was developed by CS and discussed with AP. CS reviewed the instruments using the code book and excerpts of the instruments that related to the codes was inserted into a spread sheet. CS and AP then analysed the instruments according to these codes.

## Results

### Consent

The importance of informed consent was stressed in all instruments. The following discussion is on informed consent as an ethical requirement in research, as distinct from consent as a legal basis for processing personal data or special personal data.

The need for informed consent was discussed in the context of the collection of biological samples and data, the use of biological samples and data in research, and the secondary use of these samples and data for research. A refusal to consent to the removal, storage or use of biological materials or data should not result in discrimination of the participant (2016 CoE). Broadly, the discussion on informed consent covered five aspects: 1) consent must be prior, free and informed; 2) differing models of consent; 3) consent must provide for a withdrawal of consent; 4) circumstances when consent could not be obtained due to incapacity; and 5) community consultation. We will consider each of these in turn.

To be informed, participants must be told about the scope of the research, foreseeable risks and potential benefits, but the benefits must not be presented in a way that could be construed as an improper inducement to the research. This should include a discussion of the biological sample and data to be collected, data to be derived from the sample, whether any access to the participants health records is required, the intended use of the sample and data, where and how the sample will be stored and protected, the various policies related to the sample and data (see below on policies), the governance procedures of the biobank and/or databank, and any rights or safeguards provided by law. This informed consent should be documented, preferably in a signed document.

The need for specific consent in research was stressed by many of the instruments (e.g. 2019 CoE stated that “in principle” consent must be the basis for processing health data unless provided for by law), but broad consent was either implicitly or explicitly permitted by many of the instruments. Data and samples may be provided for particular research projects through specific consent, or for multiple uses under broad consent, depending on its status in national laws. The broadness of the broad consent does vary throughout the instruments. The 2016 CoE states that participants should be provided with information that is “as precise as possible”, whereas Taipei permits storage in a biobank for “multiple and indefinite uses”.

The instruments generally permit the secondary use of samples and data without re-contacting participants, if certain conditions are fulfilled. This could occur: 1) where broad consent is the legally accepted consent model and permits the re-use of samples and data; 2) where it would be impossible to obtain consent and this could impair the research; 2) where it would be impracticable or not compatible to achieve the purpose of the research; and 4) participant had not previously objected to such research use. Generally, the use of broad consent is only permitted after independent review. Some instruments stressed that an individual retains the right to request information about their data use, and measures should be put in place to ensure that such requests can be honored.

Specific informed consent, broad consent, and waivers of consent were the only consent frameworks specifically mentioned. However, the 2016 CoE did note that participants should be told about “the possible choices that he or she could exercise”. This could perhaps be a recognition of tiered consent.

Some of the instruments also spoke of the use of residual samples stored after “past research, clinical or other purposes” (as per CIOMS). Consent would not have been obtained for future, unspecified research, and “reasonable” efforts should be made to re-contact the research participant. Where this is not possible, a waiver of consent is possible, subject to independent approval.

At the time of consent, participants must be informed of their right to withdraw consent, the procedure for doing so, and should not be subject to any form of discrimination for exercising their right to withdraw. If a participant exercises this right to withdraw their consent, they should have the option to have their sample and data returned to them, destroyed, or rendered anonymous. The future use of this sample and data is not permitted after the withdrawal of consent. There can be no penalty or negative consequences as a result of this withdrawal. Despite the importance of the withdrawal of consent as part of informed consent, many of the instruments provide for limitations on withdrawal of consent (2009 OECD, 2016 CoE). In such cases, participants must be told during the informed consent process if there are any limitations on the withdrawal of consent. What is lacking is guidance as to when limitations on withdrawal of consent could be justified, other than that they should be in accordance with the ethical and legal principles of that country.

The instruments provided for when consent could not be obtained due to lack of capacity. In such circumstances, consent can either be obtained from a substitute decision maker (2009 OECD Guidelines, Declaration of Helsinki) or a waiver of consent can be obtained from a REC or appropriate authority (2009 OECD Guidelines), but this should be provided by law (2016 CoE Recommendation).

Three instruments discussed community consultation, community interest and community consent. The Declaration of Helsinki noted that it may be important or necessary to “consult” family members or community leaders, and the 2005 UNESCO noted that it may be appropriate to obtain consent from community representatives where research is to be carried out on a community. Taipei states that the interests and rights of the communities concerned, particularly when vulnerable, must be protected. However, this community consent must be in addition to and not a substitute to individual informed consent. CIOMS also encourages researchers, sponsors and health authorities to engage with participants and communities throughout the life-cycle of the research.

### Independent Review and Oversight

The instruments broadly discussed four differing types of review and oversight: 1) review and oversight in the establishment of a biobank and databank; 2) review and oversight of the research; 3) ethical review of the research; and 4) review and oversight of the secondary use of samples and data. For all, the review must be independent.

The 2016 CoE requires review and oversight in the establishment of any biobank or databank. This review should be aimed at “safeguarding the rights and interests” of the participants and ensuring compliance with the provisions of its recommendation. CIOMS, Helsinki, and Taipei specifically require independent *ethical* review prior to the establishment of a biobank and a databank. The 2009 OCED recommend independent ethics review as best practice in the establishment of biobanks and the 1997 UNESCO encourages states to establish ethics committees to assess the ethical, legal, and social implications of research on the human genome.

The need for independent review and oversight is essential for research generally. Similar to the establishment of biobanks, there is a distinction between independent review that appears to encompass ethical review, and instruments that require a distinct ethics review. The Oviedo Convention, 2005 CoE and 2016 CoE requires independent oversight that includes an independent review of the scientific merits of the research project, an assessment of the aims of the research, and a review of the ethical acceptability of the research. The 2017 OECD is more nuanced, noting the importance of “multidisciplinary review”, and, similarly, 1997 UNESCO requires review in accordance with national and international guidelines and standards. The 2005 CoE does consider ethics review (Chapter III), and this seems to be distinct from the independent examination in Article 7.

This independent review should also consider the secondary use of samples and data (e.g. 2016 CoE), and the Helsinki and Taipei Declaration require REC review prior to the use and reuse of samples in research. The 2003 UNESCO recommends consulting ethics committee in the collection, storage and use of biological materials “where appropriate”. The 1997 UNESCO is more nuanced, encouraging states to establish multidisciplinary ethics committees to “assess the ethical, legal and social issues raised by research on the human genome and its applications”.

Overall, it would appear that there must be independent review in the establishment of a databank or biobank and use of samples and data in research. Conversely, only some require independent review in the secondary use of samples and data in research.

### Accountable Processes

It was clear from the instruments that genetic and biobank research can only proceed with accountable processes in place to govern access and use of samples and data. The 2009 OECD recommends that biobanks should be established and operate in accordance with “applicable legal frameworks and ethical principles”, a point echoed in its 2017 Recommendation. The 2003 UNESCO Declaration also recommends that states consider establishing a framework for the “monitoring and management” of human genetic data. Taipei encourages the development of laws and policies protecting biological materials and data.

At a more local level, the instruments require governance processes, with clear lines of accountability in the collection, use, re-use, and sharing of samples and data. These lines of accountability and the governance structure must be made public. An individual should be appointed with the responsibility for the security and privacy of the collections, as well as informing relevant individuals about their legal duties and responsibilities in relation to the sample and data use.

Linked to this are issues of access by third parties (as distinct from access by the research participant). The 2009 OECD, the 2016 CoE and CIOMS guidelines discuss in detail access to samples and data. Access requests should be subject to independent review and must include a research plan that is ethically and scientifically robust. Upon approval of any access requests, the transfer of samples and data should be accompanied by a legal agreement between the sender and the recipient of the samples and data. This legal agreement should include the consent or authorization on the use of the samples and data, any restriction on use as specified by the participant in their consent, the data or sample that the recipient is getting access to, the necessary arrangements for the secure transfer of the data, the duration of the sample and/or data use, and what is to happen to the samples and/or data after they are returned. The samples and data must be documented in such a way that it can be retrieved. Importantly, the responsibilities of all parties must be specified, along with the sanctions in the event of non-compliance.

### Clear and Transparent Policies and Processes

A cross-cutting issue throughout the instruments is the need for clear and transparent policies on all aspects of the collection, use and management of the samples and data, including secondary uses. The policies required did vary according to the instrument, but they included policies on consent, retention and storage, accessing data from other sources, linking of the data with other datasets, access requests by third parties in line with the participants informed consent, access requests by the research participant, benefit sharing, and feedback of results and findings. With the exception of the access policy, the instruments did not detail the content of the policies, rather focused on what policies should be developed. These policies and governance structures must be made publicly available.

In addition to the policies themselves, there should be information on the research conducted. The research participant must be provided with the information specified as part of informed consent and, in addition, they must be informed of any data breach. However, the instruments also point to the obligation to inform the general public. There should be public information on the research itself, the goals and objectives of the research, the type of data held, and aggregated research findings should be made available. Any sources of funding must be disclosed publicly. A catalogue of the resources available for research purposes must be made available.

### Security

The instruments were all cognizant of the need to secure the data, the importance of security as a key protection in the preservation of privacy, and that this is the responsibility of those processing the data. However, CIOMS also requires the REC to review the security arrangements and the Declaration of Taipei requires security arrangements to be detailed as part of the governance structure.

The instruments discussed technical measures to secure the data and samples and procedural measures to secure the data and samples. Most instruments are clear that the research participants are not to be identified. Some did state that where possible, the data be anonymized, but the coding or de-linking of the sample and data is a preferred option as this enables the retrieval of the sample and data after it has been shared.

The 2017 OECD guidelines highlighted coding and encryption of the data, data enclaves, secure data access centers, systems to verify and authenticate those accessing the samples and data, and logging of access to the samples and data as possible security measures. Outside of this, the remaining instruments simply spoke of the need for some privacy preserving security measures and processes to be put in place to prevent unauthorized access to the data and samples. The transfer of data and samples cannot take place without evidence that the recipient has adequate security measures in place. Furthermore, all processes and protocols aimed at preserving the privacy and security of the research participants should be documented.

### Training and Education

The need for training and education of those who are handling the biological samples and data was highlighted in some of the instruments. First, those conducting the scientific research must have the necessary technical skills required for the research. Second, individuals processing biological samples and data require training in privacy and security and this training should be in line with best practice and any relevant professional codes of practice, and the training must be commensurate with the roles and responsibilities of the individual (e.g., 2017 OECD). Third, the need for ethics training was also highlighted (CIOMS and 2003 UNESCO). REC members must receive the necessary ethics training to fulfil their role but, this ethics training must extend beyond REC members and include researchers.

Irrespective of the type of training, it should be ongoing. There was also the suggestion that an individual should be appointed for ensuring compliance with the relevant security and privacy standards, as well as updating on the legal obligations related to the sample and data use.

## Discussion

The review of the instruments identified consent, independent review and oversight, accountable processes, clear and transparent policies and processes, security, and training and education as possible safeguards for biobank, databank, and genetic research. Some instruments discussed possible risks such as (in addition to privacy), risks of stigmatisation and discrimination, and the need to mitigate against those risk. A necessary first step in the consideration of possible safeguards is a risk assessment, and the GDPR itself requires a risk assessment in advance of high-risk processing. Risk to the participant must not just consider the data itself, but also what can be inferred from the data. This will require a consideration of the type of data being used as well as potential uses of the data and samples and their analysis and from this assessment safeguards to mitigate against these risks can be selected. There are four pertinent points to be made on this assessment. First, this risk assessment should be done in advance of the establishment of a biobank, a databank, the use, *and* secondary use of the samples and data. This will ensure that privacy by design is embedded as part of the research. Second, due to the importance of transparency both within the instruments and the GDPR itself, a risk assessment that identifies the risks, mitigation strategies and any residual risks should be included as part of each research protocol. Third, this risk assessment is to be considered to be a living document that must be regularly reviewed throughout the life-cycle of the project and in accordance with changes in technology. Fourth, the data controller must demonstrate that the safeguards identified are appropriate in the context of the research, in accordance with the GDPR, and ensure respect for data minimization. On this last point, the instruments do provide some additional guidance as to possible safeguards, but perhaps the most attention is given to consent, accountable processes that include independent review, and the policies that must be put in place.

Overall, there is a preference for specific consent, with an acceptance of broad consent if certain conditions are met. Similarly, a waiver of consent is permitted if there is ethical review. Other consent models, such as tiered consent, are not explicitly considered, and dynamic consent is a notable absence from the instruments. Howver, in the context of the GDPR, with the exception of Member States that require consent for the processing of genetic data (such as Italy), consent may not be the legal basis selected to process personal data for research (Dove and Chen, 2020). In such circumstances, the EDPB has stated that informed consent may be considered to be a safeguard if it is “a means for giving individuals more control and choice and thereby for upholding society’s trust in science”. It is important that there is an understanding between *informed consent* as an ethical requirement in research and *consent* as a legal basis for the processing of personal data and that the correct terminology (i.e. informed consent *vis a vis* consent) is used to avoid any confusion (Gefenas et al., 2021).

The various consent models specified in the instruments (specific consent, broad consent, and waiver of consent) and those not specified in the instruments (tiered and dynamic consent) have been adopted for biobank research, databank research, and genetic research and thus are appropriate for this research. Specific consent is preferred under the various instruments. This provides the research subject with control and choice over the use of their data in research and thus would be in accordance with the GDPR.

Outside of specific informed consent, broad consent is permitted within the instruments. Recital 33 of the GDPR states that research participants can give their consent to the use of their data “to certain areas of scientific research” and it is thought that broad consent is permitted under the GDPR (Hallinan, 2020). However it seems to be a narrower conceptualization of broad consent than that of some instruments, which appear to permit the collection for future use in research generally. The question, thus, is whether this wider concept of broad consent can be considered a safeguard when a lawful basis other than consent is used for the processing of personal data for research. We would argue that it is, provided that broad consent is demonstrated as being the most appropriate consent model for the research, there is independent review of the use of broad consent, and the future use of the data is subject to independent review; in other words: provided there are additional safeguards in place to uphold the rights of the research participants. This provides for a transparent and accountable process in the use and re-use of data in research, and also provide for an independent body to ensure that no more data than is needed is provided to the researcher, therefore ensuring respect for the principle of data minimization. We, therefore, endorse and agree that broad consent is “consent for governance” (Koenig, 2014; Tindana and de Vries, 2016), and that it should only be adopted after an appropriate body has reviewed the compatibility of the existing consent with any new research purposes.

Although not explicitly mentioned in the instruments, tiered consent and dynamic consent should be considered. Tiered consent provides the research participant with a range of options on which to consent to (and therefore more aligned with the requirements of Recital 33), of which one option may be broad consent. This consent model thus provides the research participant with more choice and control. Similarly, dynamic consent enables donors to narrow broad consent based on individual preferences (Kaye et al., 2015). It facilitates active participation in research, empowers individuals to control and determine how and where their samples and data should be used, to be kept informed about the uses of their data and samples and to timely object to further uses or to withdraw when circumstances change their willingness to be part of the research (Kaye et al., 2015; Mamo and Martin, 2020; Biasiotto et al., 2021). Both consent models give more control to the research participant and would be in accordance with the GDPR. Under both models, the re-use of the data in research will be subject to independent review and thus considerations of data minimization should be had at this review stage.

The instruments do state that the withdrawal of consent must be respected but acknowledge that there are limitations. However, such limitations on the withdrawal of consent are unlikely to be permitted where consent is the legal basis for the processing of personal data.

Consent is clearly a safeguard that should continue to be embedded in the use and secondary use of data for research. However, it is important that the governance of the consent model adopted ensures respect for the rights of the research principles.

Linked to consent, there is a clear need for independent review in the instruments, and this is discussed to varying degrees in the context of collection, use, and re-use of the data. The EDPB has noted that researchers operating within an ethical framework should be able to access data, provided there is a valid legal basis and subject to safeguards. Independent and multidisciplinary review is a long-standing requirement in the ethical conduct of research and is, therefore, appropriate in this context. There are differing stages of independent review that must be looked at: (i) the establishment of the biobank or databank, (ii) the use of the samples in research, (iii) and any future use. This independent review could be considered to be an organizational measure that is required as part of the establishment of a biobank and the use and re-use of any personal data in research and can consider the risks to the participants, efforts to mitigate against such risks, and how their rights and freedoms will continue to be safeguarded in the research. It can also importantly consider whether the biobank or the researcher requires all the personal data requested and if it is in line with the principle of data minimization. The independent review could follow that guidance of the EDPB that has stated that this can be achieved through specifying the research question and assessing the type and amount of data necessary to answer the research questions. Data controllers and Member State law should therefore require independent review in advance of the establishment of a biobank, and prior to the use *and re-use* of personal data in research. This is particularly important in the use of a broad and tiered consent model. The research participant is handing over control of their personal data, but under the understanding that there is appropriate governance in place. This independent check can verify that the secondary use continues to respect their fundamental rights and freedoms. While we see independent review a key safeguard in biobank, databank, and genetic research, it is important that data controllers remember that they are responsible for demonstrating compliance with the GDPR under Article 5(2) and that this independent review does not absolve them of their obligations or guarantee compliance with the GDPR.

Throughout the instruments it is clear that the governance of the research is critical and there is a clear need for certain policies to be put in place. Such policies are increasingly common in biobank, databank, and genetic research and, thus, are appropriate. Overall, the instruments generally did not mandate what the policies should state, rather only required that policies on the various topics are in place. Such policies will include the transparency of data use and provide for accountable measures, and, as such, are in accordance with the GDPR.

The policy that receives the most attention within the instruments is an access policy, unsurprising considering the possible risks to the data subjects in the sharing of their personal data for research. An access policy must specify the requirements to be followed for access requests, a research plan must be included in any access request, and access should only be provided subject to a legal agreement. Most biobanks do require specific conditions for granting access to their data and samples, and access is dependent on provision of details on research aims, but it is not always stipulated explicitly whether the scientific merit of access requests is screened (Capocasa et al., 2016). Many biobanks have specific access committees evaluating access requests and adjudicating access arrangements, with a mandate to strike the balance between protecting participants and maximizing the use of the biobank (Fortin et al., 2011).

Outside of the access policy, in general, the instruments require policies on consent, data and sample retention, security, and the governance of the biobank. The policies on consent should specify the consent model to be used, the right to withdraw consent and any limits on the right to withdraw (Duguet and Herveg, 2021). The principles of transparency, accountability, and privacy by design would require such policies to be developed in advance of the collection and use of data and that these policies should be publicly available. However, a 2017 review of 523 biobanks found that only 9% of them had publicly available access policies (Langhof et al., 2019). What is emerging is the need for clear, transparent, and publicly available policies on all aspects of the use and re-use of personal data in research. .

Although many of the instruments discuss the importance of security, but do not provide much detail. Overall, the discussion on the technical measures in securing data was general in nature, focusing more on what *they should do* (i.e., secure the data and samples) and less on *how* that should be achieved. It is worth noting it is the persons processing data that are often the weak link in data security, therefore the training of all those handling the personal data will be key (Anderson et al., 2020). It is appropriate that the research team have training in security and privacy and Article 39(1)(b) designates the data protection officer with responsibility of training any staff involved in the processing of personal data. Adequate training of all staff would also be important in demonstrating compliance with the principles of the GDPR as required under Article 5(2). As part of this training, staff could be trained on how they can ensure respect for the principle of data minimization. However in addition to training on security and privacy, research staff should also receive ethics training on the importance of the ethical conduct of research and the safeguarding of the rights of the research participant and the principles of data protection.

## Conclusion

The GDPR provides derogations from many of the strict processing requirements, but the applicable of all derogations may leave research participants with limited rights under the GDPR. Such derogations are only legitimate if the processing is for research, if reliance on the derogations are necessary for the research, and the derogations comply with Article 89. Under Article 89, it is essential that appropriate safeguards are adopted, but there is limited guidance within the GDPR on what could be considered to be appropriate safeguards. The much-anticipated Opinion from the EDPB should provide some guidance and this is something that needs to be regulated with the EHDS regulation in greater detail. The appropriateness of safeguards will depend on the research, and the context in which the research takes place. While we await such guidance, in the interim, our study has identified six possible safeguards for biobank, databank, and genetic research: consent that is appropriately governed; independent review and oversight; accountable processes; clear and transparent policies; adoption of security measures; and training and education of all of those involved in the use and re-use of personal data in research. We argue that these safeguards must not only apply to the collection and use, but also to subsequent re-use of personal data in research. In this way, it will provide for an integrated bioethics approach to data protection and research.

## References

[B1] Anderson D. Abiodun O. P. Christoffels A. (2020). Information Security at South African Universities-Implications for Biomedical Research. Int. Data Privacy L. 10, 180–186. 10.1093/idpl/ipaa007

[B2] Biasiotto R. Pramstaller P. P. Mascalzoni D, (2021). The Dynamic Consent of the Cooperative Health Research in South Tyrol (CHRIS) Study: Broad Aim within Specific Oversight and Communication. BioLaw J. - Rivista Di BioDiritto 21, 277–287. 10.15168/2284-4503-786

[B4] Capocasa M. Anagnostou P. D’Abramo F. Matteucci G. Dominici V. Destro Bisol G. (2016). Samples and Data Accessibility in Research Biobanks: An Explorative Survey. PeerJ 4, e1613. 10.7717/peerj.1613 26966643 PMC4782685

[B5] Dove E. S. Chen. J. (2020). Should Consent for Data Processing Be Privileged in Health Research? A Comparative Legal Analysis. Int. Data Privacy L. 10 (2), 117–131. 10.1093/idpl/ipz023

[B6] Duguet A.-M. Herveg J. (2021). “Safeguards and Derogations Relating to Processing for Scientific Purposes: Article 89 Analysis for Biobank Research,” in GDPR and Biobanking: Individual Rights, Public Interest and Research Regulation across EuropeSanta Slokenberga, Olga Tzortzatou, and Jane Reichel (Cham: Law, Governance and Technology SeriesSpringer International Publishing), 105–120. 10.1007/978-3-030-49388-2_7

[B7] EDPB (2020a). Guidelines 03/2020 on the Processing of Data Concerning Health for the Purpose of Scientific Research in the Context of the COVID-19 Outbreak | European Data Protection Board. Available at: https://edpb.europa.eu/our-work-tools/our-documents/guidelines/guidelines-032020-processing-data-concerning-health-purpose_en (Accessed June 1, 2021).

[B8] EDPB (2020b). ‘Preliminary Opinion on Data Protection and Scientific Research | European Data Protection Supervisor’. Available at: https://edps.europa.eu/data-protection/our-work/publications/opinions/preliminary-opinion-data-protection-and-scientific_en (Accessed June 1, 2021).

[B9] Fortin S. Pathmasiri S. Grintuch R. Deschênes M. (2011). ‘Access Arrangements' for Biobanks: A Fine Line between Facilitating and Hindering Collaboration. Public Health Genomics 14, 104–114. 10.1159/000309852 20689244

[B10] Gefenas E. Lekstutiene J. Lukaseviciene V. Hartlev M. Mourby M. Cathaoir K. Ó. (2021). Controversies between Regulations of Research Ethics and protection of Personal Data: Informed Consent at a Cross-Road. Med. Health Care Philos. 10.1007/s11019-021-10060-1 PMC8595272 34787769

[B11] Hallinan D. (2020). Broad Consent under the GDPR: An Optimistic Perspective on a Bright Future. Life Sci. Soc. Pol. 16, 1. 10.1186/s40504-019-0096-3 PMC6943899 31903508

[B12] Kaye J. Whitley E. A. Lund D. Morrison M. Teare H. Melham K. (2015). Dynamic Consent: a Patient Interface for Twenty-First century Research Networks. Eur. J. Hum. Genet. 23 (2), 141–146. 10.1038/ejhg.2014.71 24801761 PMC4130658

[B13] Koenig B. A. (2014). Have We Asked Too Much of Consent? Hastings Cent. Rep. 44, 33–34. 10.1002/hast.329 PMC4249719 25043364

[B14] Langhof H. Schwietering J. Strech D. (2019). Practice Evaluation of Biobank Ethics and Governance: Current Needs and Future Perspectives. J. Med. Genet. 56 (3), 176–185. 10.1136/jmedgenet-2018-105617 30464052

[B15] Mamo N. Martin G. M. Desira M. Ellul B. Ebejer J.-P. (2020). Dwarna: a Blockchain Solution for Dynamic Consent in Biobanking. Eur. J. Hum. Genet. 28 (5), 609–626. 10.1038/s41431-019-0560-9 31844175 PMC7170942

[B16] Slokenberga S. (2021b). You Can’t Put the Genie Back in the Bottle: On the Legal and Conceptual Understanding of Genetic Privacy in the Era of Personal Data Protection in Europe. BioLaw J. Rivista Di BioDiritto 21, 223–250. 10.15168/2284-4503-783

[B17] Slokenberga S. (2021a). ‘Setting the Foundations: Individual Rights, Public Interest, Scientific Research and Biobanking’. GDPR and Biobanking, 11–30. 10.1007/978-3-030-49388-2_2

[B18] Staunton C. Slokenberga S. Mascalzoni D. (2019). The GDPR and the Research Exemption: Considerations on the Necessary Safeguards for Research Biobanks. Eur. J. Hum. Genet. 27, 1159–1167. 10.1038/s41431-019-0386-5 30996335 PMC6777499

[B19] Tindana P. de Vries J. Campbell M. Littler K. Seeley J. Marshall P. (2015). Community Engagement Strategies for Genomic Studies in Africa: A Review of the Literature. BMC Med. Ethics 16, 24. 10.1186/s12910-015-0014-z 25889051 PMC4407431

[B20] Tindana P. de Vries J. (2016). Broad Consent for Genomic Research and Biobanking: Perspectives from Low- and Middle-Income Countries. Annu. Rev. Genom. Hum. Genet. 17, 375–393. 10.1146/annurev-genom-083115-022456 26905784

